# Solid Dosage Forms of Dexamethasone Sodium Phosphate Intended for Pediatric Use: Formulation and Stability Studies

**DOI:** 10.3390/pharmaceutics12040354

**Published:** 2020-04-14

**Authors:** Maria S. Synaridou, Eleftherios G. Andriotis, Constantinos K. Zacharis, Dimitrios G. Fatouros, Catherine K. Markopoulou

**Affiliations:** 1 Laboratory of Pharmaceutical Analysis, Department of Pharmacy, Aristotle University of Thessaloniki, 54124 Thessaloniki, Greece; msynarid@pharm.auth.gr (M.S.S.); czacharis@pharm.auth.gr (C.K.Z.); 2 Laboratory of Pharmaceutical Technology, Department of Pharmacy, Aristotle University of Thessaloniki, 54124 Thessaloniki, Greece; adrioti@pharm.auth.gr (E.G.A.); dfatouro@pharm.auth.gr (D.G.F.)

**Keywords:** chocolate formulation, dexamethasone sodium phosphate (DSP), high-performance liquid chromatography (HPLC), in vitro digestion

## Abstract

Undesirable taste has always been a key issue for oral dosage forms. The aim of the present study was to co-formulate dexamethasone sodium phosphate (DSP), in common pediatric oral forms, using sweet preserves and/or different types of chocolate as excipients. An array of different kinds of chocolate were co-formulated with DSP and were further characterized by means of dynamic light scattering (DLS), x-ray diffraction (XRD), differential scanning calorimetry (DSC) and Fourier-transform infrared (FT-IR) spectroscopy. For the assay of active pharmaceutical ingredient (API), the chocolate samples were pre-treated by means of liquid extraction and analyzed using an high-performance liquid chromatographic (HPLC) method with a strong anion exchange column and a phosphate buffer (17 mM, pH = 3)/acetonitrile, 50:50 *v*/*v* as mobile phase. The developed chromatographic method was validated based on the International Conference on Harmonization (ICH) guidelines (%Mean Recovery = 99.4% and %Relative Standard Deviation, RSD = 0.43%). Furthermore, dissolution and in vitro digestion tests of chocolate formulations were evaluated. The DSP was found to be stable for at least 1 year in prepared preparations.

## 1. Introduction

Taste is considered to be the main sensory system which affects the acceptability of any medicine, especially in oral dosage form. Some patients, particularly children, are reluctant to take certain types of medicines mainly due to their unpleasant organoleptic properties, such as smell, taste, aftertaste, texture, etc. [1]. Therefore, there is a need to improve the palatability of pediatric pharmaceutical products and this issue has become a key aspect of modern pharmaceutical development studies [2,3].

Corticosteroids are naturally occurring compounds produced by the adrenal gland in the body and are involved in a wide range of physiological processes. Dexamethasone sodium phosphate (DSP) is a glucocorticosteroid utilized for the treatment of many inflammatory conditions, such as allergic disorders, ulcerative colitis, arthritis, psoriasis, etc. According to the World Health Organization (WHO), DSP is considered to be one of the most effective and safest medicines [4,5] for a lot of therapeutic schemes. In the human body, DSP is hydrolyzed rapidly by phosphatases producing its free form [6].

Owing to its low water-solubility, dexamethasone, the commercially available formulation in Greece namely Soldesanil^®^, is an oral liquid drops solution containing dexamethasone as a salt solution in dexamethasone sodium phosphate (DSP) form. Unfortunately, the bitter taste of the DSP results in poor patient compliance. Taste-improving techniques can be helpful to overcome such issues by producing either new palatable formulations or by covering the unpleasant flavor with food matrices. Among other parameters, the stability of DSP in such formulations plays a vital role and should be thoroughly investigated. Similar trials have also been carried out in our lab, but in none of them are the active pharmaceutical ingredients (API’s) incorporated in the palatable matrices [7].

**Figure 1:**
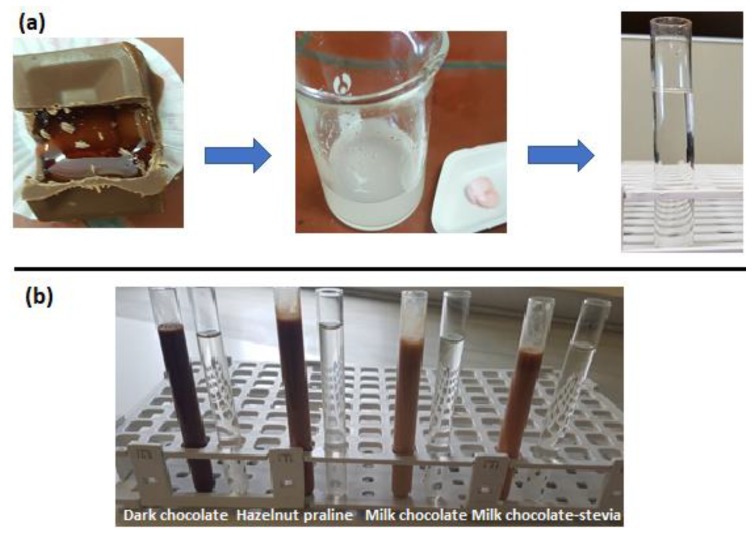
Procedure before and after cleaning-extraction. (**a**) Chocolate-strawberry syrup formulation (**b**) dark chocolate, hazelnut praline, milk chocolate, milk chocolate—stevia.

In the current study, we detail alternative ways of delivering dexamethasone sodium phosphate as an active ingredient, without losing its potency and stability. Hence, the drug’s taste might be improved either by mixing the appropriate quantity of Soldesanil^®^ drops with a strong, sweet-flavored matrix, like hazelnut praline and strawberry or cherry syrup, or by administering the new pharmaceutical preparation with chocolate and DSP. Moreover, a fast and reliable high-performance liquid chromatography–ultra-violet spectroscopy (HPLC-UV) method has been developed and validated for the determination of DSP in the prepared formulations (Figure 1). The method was successfully applied to estimate the dissolution profile and to support the in vitro simulation of gastrointestinal digestion experiments.

On surveying the literature, there are numerous analytical approaches to determine DSP in various samples of biological, animal-derived foodstuffs, etc., in which liquid chromatography (LC), coupled with spectrophotometric (UV) [8] or mass spectrometric detection (MS), is the predominant analytical technique [9,10,11]. Concerning the sample preparation, several efforts have been made to isolate DSP from complicated matrices involving solid phase extraction [12,13], matrix solid phase dispersion [9,14], liquid phase microextraction (LPME) [15], QuEChERS (Quick, Easy, Cheap, Effective, Rugged and Safe) [16,17] and simple protein precipitation [18]. Most of them are laborious, time-consuming and require large amounts of consumables. Hence, they cannot be easily adopted by quality control (QC) pharmaceutical laboratories. To the best of our knowledge, there is no published method in the literature regarding the analysis of DSP in food-based formulations.

In the present study, an easy, quick and reliable HPLC-UV method has been developed for the determination of DSP in novel edible formulations aided for pediatric delivery. A set of foods, such as different types of chocolate, hazelnut praline and strawberry/cherry syrup, are used to produce DSP formulations without losing their potency or stability. An analytical method was applied to estimate the dissolution profile and to support the in vitro simulation of gastrointestinal digestion experiments. The proposed analytical scheme has been validated according to International Conference on Harmonization’s (ICH) guidelines.

## 2. Materials and Methods

### 2.1. Chemicals, Reagents and Materials

For the HPLC method: Methanol, acetonitrile and water were of HPLC grade and supplied by VWR chemicals (Vienna, Austria). Sodium dihydrogen phosphate monohydrate and phosphoric acid were purchased from Merck, while DSP and propylene glycol (PG) were from Sigma-Aldrich (Steinheim, Germany) and met United States Pharmacopeia’s (USP) specifications.

For the preparative method: Ingredients, such as hazelnut praline, different types of chocolate and fruit syrup, mainly of natural origin, were provided by local Greek markets and were used as excipients. The main features of the different substrate types used as a carrier of DSP were the following:

Dark chocolate (or bitter-sweet chocolate) mainly presents antioxidant properties due to its high content of flavonoids and vitamins [19]. Dark chocolate may contain fats to soften the texture (35% chocolate liquor) and dry cocoa, but it does not generally have a milky flavor.

Milk chocolate is the most commonly consumed chocolate, and according to Food and Drug Administration (FDA) regulations, must contain at least 10% chocolate liquor and 12% milk solids. Cocoa butter and milk solids are the only fat ingredients allowed in milk chocolate [20].

White chocolate is a chocolate confection made from cocoa butter, sugar and milk solids. Since it does not contain cocoa solids, which are found in other types of chocolate, only trace amounts of the stimulants theobromine and caffeine can be found.

In chocolate with stevia (low-fat chocolate), sugar has been replaced by a natural sweetener. The International Dairy Foods Association (IDFA) and the National Milk Producers Federation (NMPF) support that the use of non-nutritive sweeteners would promote more healthful eating practices and reduce childhood obesity.

Hazelnut chocolate praline (merenda) is chocolate made with hazelnuts ground into powder, cocoa and skim milk. It still has a smooth, chocolatey texture and the flavor of hazelnuts.

Sweet preserves: In Greece, they are called spoon sweets because the usual serving size is a full teaspoon. The syrup from this product contains mainly sugar, water and ingredients from the boiling of strawberries or cherries.

Soldesanil^®^-drops (10 mL) (European Medicines Agency (EMA), NAN:21266/2-4-2008) [21] pharmaceutical formulation containing DSP (2 mg/mL, 32 drops) and propylene glycol as excipient, was supplied by a local pharmacy in Greece.

For the digestion and dissolution test: For the dissolution test, a diluted hydrochloric acid (1 in 100) solution from Sigma-Aldrich (Steinheim, Germany) was prepared. For the Simulated Salivary Fluid (CSF) and the Simulated Digestion Fluids, the following reagents were used: Sodium bicarbonate (0.5 M) was prepared and filtered through a 0.22 μm syringe filter (stored at 2–5 °C for one month). All enzyme products were provided by Sigma-Aldrich (Human saliva Type IX-A, 1000–3000 U mg^−1^ protein and Pepsin from porcine gastric mucosa 3200–4500 U mg^−1^ protein, Sigma-Aldrich, Taufkirchen, Germany).

### 2.2. Manufacturing Process of Edible Formulations

Different types of chocolate or fruit syrups were used as a carrier or overcoat layer for dexamethasone sodium sulphate. They have a dual role since they eliminate the API’s deterioration and improve the overall flavor of the formulation. According to FDA guidelines, the acceptable pediatric dose of an anti-inflammatory substance is 0.02–0.4 mg/kg every 8 to 12 h. Therefore, for a child aged 2–3 years, with an average weight of approximately 13–14 kg, an average single dose between 0.3 mg to 5.6 mg is required [22]. Consequently, preparations were formulated which either contained 1 mg DSP (or 16 drops of Soldesanil^®^) or 2 mg DSP (or 32 drops of Soldesanil^®^) to achieve an optimal dispersion of the active agent in the carrier (different types of chocolate) and a series of non-aqueous solvents were tested. Ultimately, propylene glycol was chosen as the most convenient solvent. According to the European Medicines Agency (EMA), the maximum daily dose of propylene glycol (PG) that is considered to be safe for a child aged 2–3 years is 50 mg/kg (700 mg/14 kg) [23]. Thus, an appropriate quantity of DSP was mixed with PG, alternating between 5 min stirrings and ultrasonic baths for a total of 20 min (mix A). The quantities of excipients and DSP in each formulation are presented analytically in Table 1. All samples were kept at 4 °C until use. The quantities of excipients and DSP in each formulation are presented in Table 1.

#### 2.2.1. Chocolate-Based Dexamethasone Sodium Phosphate (DSP) Formulations

4 g of different types of chocolate (milk, dark, white or milk with sweetener) were melted at 60 °C in a water bath and mixed with the appropriate portions of Mix A in order to produce DSP formulations of 1 or 2 mg, respectively (Table 1). The mixture was homogenized and quantitively transferred to a pre-weighed silicone mold, greased with butter, (4 × 2 cm, Frigo Hellas serial number: 66801). Finally, the formulations were placed in the refrigerator (−18 °C) for about 3 h to obtain a solidified form.

#### 2.2.2. Hazelnut Praline Alternative

16 drops of Soldesanil, equal to 1 mg of DSP, were mixed with 4 g of hazelnut praline (merenda).

#### 2.2.3. Fruit Syrup/Chocolate-Based DSP Formulations

The proposed formulations consist of a core containing syrup from sweet preserves, in which the active ingredient dexamethasone sodium phosphate was encapsulated. Sequentially, the core was coated with milk chocolate.

According to the procedure, 4 g of strawberry or cherry syrup were mixed with Mix A to produce the core of the formulation. Subsequently, the core was coated with chocolate. To prepare the chocolate coating, an amount of 4 g of milk chocolate was melted at 60 °C in a water bath and placed in a pre-weighed silicone mold to create a film (ca 1 mm thickness). After the solidification of the chocolate-based film, the core was quantitively transferred onto the chocolate base and covered with a thin layer of chocolate. The final DSP-formulation was placed in the refrigerator (−18 °C) for about 3 h.

### 2.3. Preparation of Standard Solutions

A standard stock solution of DSP (1000 μg mL^−1^) was prepared by dissolving the appropriate amount of the substance in water. Subsequentially, working standards (8–64 μg mL^−1^) were made by adding appropriate dilutions of the stock solution into a mixture of acetonitrile-H_2_O, 80:20 *v*/*v*. All solutions were kept at −18 °C.

### 2.4. Samples Pretreatment

Samples (chocolate, hazelnut praline) containing 1 or 2 mg DSP were placed in a water bath at 60 °C for 15 min to reduce viscosity. Following that, 15 mL of H_2_O were added (or 45 mL in the case of hazelnut praline) and the mixture was homogenized by stirring and then sonicated for 15 min. An aliquot of 2 mL of the resulting solution was transferred into a 10 mL volumetric flask and 8 mL of acetonitrile was added. The mixture was kept at −18 °C for 30 min to facilitate efficient protein precipitation. The supernatant was transferred into a disposable tube and centrifuged at 4850 rpm for 10 min. Finally, an aliquot of the supernatant was transferred to an HPLC vial and analyzed. In the case of the chocolate with the syrup core and API, since the formulation was crushed, the core was removed from the chocolate cover using 15 mL of H_2_O and the extraction procedure followed as described above using methanol instead of acetonitrile (Figure 1).

### 2.5. Instrumentation and Method of Analysis

#### 2.5.1. High Performance Liquid Chromatography (HPLC)

Chromatographic analyses were performed using a Shimadzu HPLC system consisting of two LC-20AD isocratic pumps, a DGU-14A degasser, an SIL-10AD autosampler and an SPD-M20A diode array detector. LC-solution^®^ software version 1.25 SP4 was utilized for hardware control and data manipulation. Prior to the analysis of the samples, the system was equilibrated for approximately 20 min.

The analyte of interest was separated isocratically on a silica-based quaternary ammonium bonded (Spherisorb 250 × 4.6 mm internal diameter, 10 mm, Waters) analytical column using a mixture of phosphate buffer (17 mM, pH = 3)/acetonitrile, 50/50 *v*/*v*. The flow rate was kept constant at 1.8 mL min^−1^ while the injection volume was 20 μL. The column was thermostated to 25 °C. The DSP was detected at 240 nm.

#### 2.5.2. Dynamic Light Scattering and ζ-Potential Studies

Dynamic light scattering (DLS) and ζ-potential measurements were performed using a Malvern Nanosizer ZS, Malvern Instruments (Malvern, UK). The particle size, polydispersity index (PDI) and ζ-potential values of plain and drug-loaded samples were measured in gastric fluids, in triplicates.

#### 2.5.3. X-Ray Diffraction (XRD)

The solid-state properties of DSP with and without chocolate formulation were evaluated with an XRD analysis. Diffractograms were recorded on a Bruker D8-Advance diffractometer (Bruker AXS GmbH, Karlsruhe, Germany) using Cu Kα radiation (λ = 1.5421 Å), operated at a voltage and current of 40 kV and 40 mA, respectively. Samples were scanned over the 2θ range of 3–50° at a rate of 0.35 s/step, with a step size of 0.02°.

#### 2.5.4. Differential Scanning Calorimetry (DSC)

The thermal properties of the samples with and without DSP were characterized with differential scanning calorimetry (DSC) using a DSC 204 F1 Phoenix apparatus (Netzsch, Selb, Germany). 10 mg of the sample was loaded onto an aluminum plate and a DSC thermogram was acquired from 15 °C to 80 °C at a heating rate of 10 °C/min in a nitrogen atmosphere (70 mL/min).

#### 2.5.5. Fourier-Transform Infrared Spectroscopy (FT-IR)

FT-IR spectra of the samples were recorded using an IR Prestige-21 spectrometer (Shimadzu, Kyoto, Japan) at a range between 4000 cm^−1^ and 450 cm^−1^ with a resolution of 4 cm^−1^ after 64 scans. Samples with and without chocolate were grated into small pieces and deposited directly onto the crystal sensor.

## 3. Results and Discussion

### 3.1. Sample Pre-Treatment Procedure for HPLC Analysis

In this study, we used food-based excipients (chocolate, fruit syrup) for the preparation of edible DSP formulations. However, these matrixes are quite complicated since they contain cocoa, polysaccharides, monosaccharides, fruit extracts, fats, proteins, emulsifiers, etc. [24,25]. Due to their complexity, an efficient and reliable sample pre-treatment protocol must be developed for the isolation of the DSP drug.

**Figure 2:**
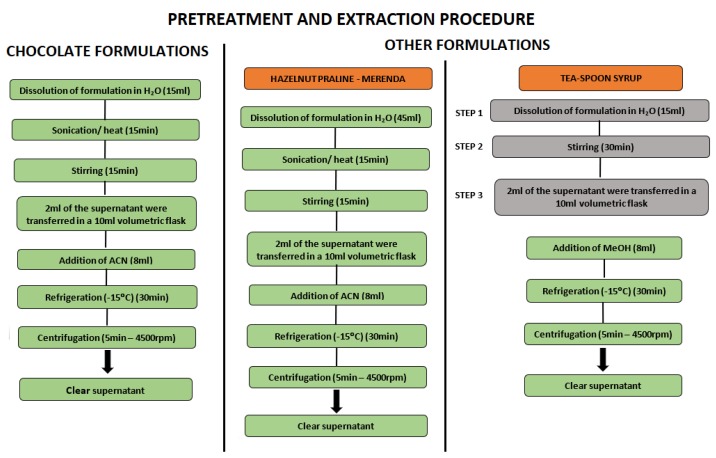
Schematic diagram of sample pre-treatment and extraction efforts.

The protein precipitation methodology, which is a low-cost, effective, simple and rapid approach, was followed. In order to achieve the optimum DSP recovery, various experimental protocols have been tried by studying the stirring time, temperature, the kind and volume of diluent, the centrifugation time, etc. The experiments revealed that acetonitrile was the most appropriate solvent for the protein precipitation of chocolate-based matrixes, resulting in a clear final extract (see Figure 2). In the case of syrup-based formulations, methanol proved to be the optimum dilution medium for sugar precipitation. Τhe optimum sample preparation conditions are presented schematically in Figure 2, whereas the unsuccessful trials are depicted in Figure S1 (Supplementary Materials).

### 3.2. Development of HPLC Conditions

Ion-exchange stationary phases are an interesting alternative when it comes to the analysis of ionizable compounds in complex matrices. Compared to the reversed phase materials, they exhibit higher selectivity and are typically less prone to hydrophobic interferences from the sample matrix, expanding the life span of the column [26]. Furthermore, the usage of organic solvents in the mobile phases is surely not excluded in ion-exchange chromatography since they broaden the separation possibilities.

In the present study, a quaternary ammonium-based strong anion exchange (SAX) stationary phase was proven superior compared to the tested hydrophobic ones, namely C_18_ and Phenyl, in terms of the retention factor and the peak symmetry of the DSP peak. Critical parameters—such as mobile phase pH, phosphate buffer concentration, acetonitrile volume fraction—affecting the chromatographic behavior of DSP were investigated.

The concentration of phosphate buffer (pH = 3.0) was examined in the range of 5 to 100 mM. As expected, the retention factor of DSP gradually reduced when buffer concentration levels were elevated, resulting in a fast-eluted sharp peak (almost non-retained) at the highest buffer concentration. Finally, a buffer concentration of 17 mM was selected as a compromise between the analysis time and method selectivity.

The volume fraction of the organic modifier also affects the selectivity of the ion-exchange HPLC method in terms of potentially co-eluting endogenous matrix compounds. On this basis, we examined the effect of acetonitrile in the range of 20% to 60% *v*/*v*. The experiments revealed that the retention factor of DSP was slightly affected by the CH_3_CN content, while enhanced chromatographic efficiency (number of theoretical plates, *N*) was recorded at a volume fraction of 50%. This value was finally chosen providing an adequate resolution of the analyte with the matrix components.

**Figure 3:**
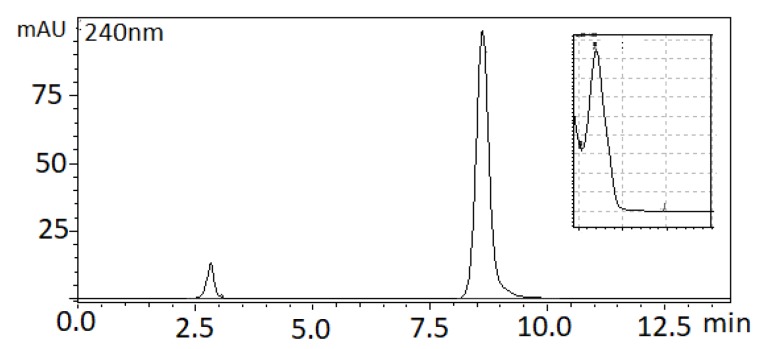
Representative high performance liquid chromatography (HPLC) chromatogram of DSP (30 μg mL^−1^).

The column temperature is a significant parameter in ion-exchange separations [24]. Increasing the column temperature from a range of 25 °C to 40 °C resulted in a decrease in the chromatographic efficiency and a slight reduction in retention time by a factor of ca 10%. The value of 25 °C was therefore chosen as the column temperature. Under the selected chromatographic conditions, the retention time of DSP was 8.5 min at a flow rate of 1.8 mL min^−1^. The analyte was monitored spectrophotometrically at 240 nm. A representative chromatogram is illustrated in Figure 3.

### 3.3. HPLC Method Validation

The HPLC analytical method was validated according to ICH guidelines in terms of linearity, selectivity, limit of detection (LOD), limit of quantitation (LOQ), precision and accuracy [27].

#### 3.3.1. Specificity

**Figure 4:**
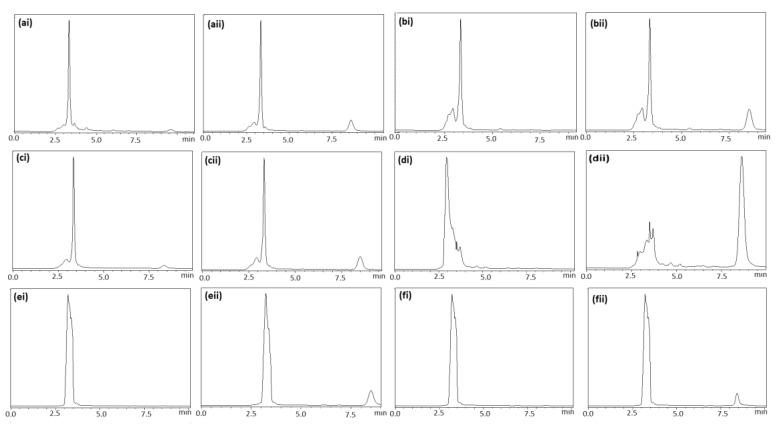
Chromatograms of blank samples (**i**) and their fortified parallel (**ii**): (**a**) hazelnut praline-merenda, (**b**) milk chocolate, (**c**) dark chocolate, (**d**) white chocolate, (**e**) strawberry syrup, (**f**) cherry syrup.

The specificity of the proposed analytical method was assessed by analyzing a set of six different placebo matrices (milk chocolate, dark chocolate, hazelnut praline, strawberry and cherry syrup) and spiked with DSP at a concentration level of 30 μg mL^−1^. All samples are treated as described in Section 2.4. As it can be seen in the chromatograms of Figure 4, the DSP was eluted as a single peak and well resolved from other peaks of the matrix that may affect the detection capability and the precision or accuracy of the method (Figure 4).

#### 3.3.2. Linearity, Limit of Detection (LOD), Limit of Quantitation (LOQ)

The linearity of the HPLC method was investigated in the range of 8.0–64.0 μg mL^−1^ by analyzing five standards. Each calibration standard was analyzed in triplicate. The linear correlation coefficient was r = 0.9994, while the regression equation was:

A(DSP) = 28,257 × γ(DSP) + 2867(1)

where A(DSP) is the area of the DSP peak and γ(DSP) is the mass concentration of DSP in μg mL^−1^. The percent residuals were distributed randomly around the “zero axis” and ranged from −5.4% to 1.7%.

The limit of detection (LOD) and quantitation (LOQ) were calculated according to the following ICH recommendations [27]:

(2) $$LOD=3.3\times \frac{S_{y/x}}{m}$$

(3) $$LOQ=10\times \frac{S_{y/x}}{m}$$

where *S_y/x_* is the residual standard deviation and m is the slope of the calibration equation. LOD values were found to be 0.14 and 0.42 μg mL^−1^, respectively.

#### 3.3.3. Precision and Accuracy

The precision of an analytical method expresses the closeness of agreement between a series of measurements obtained from multiple samplings of the same homogenous sample under certain conditions [27]. The method repeatability (intra-day precision) was assessed by analyzing six replicates of DSP standard at a 100% concentration level corresponding to 8.0 μg mL^−1^ in a single day. The intermediate precision was investigated by analyzing DSP standard at three concentration levels (8.0, 32.0 and 64.0 μg mL^−1^) on three consecutive days. Three individual replicates for each sample were carried out. Three replicates were performed at each concentration level. The relative standard deviation (%RSD) was less than 1.0% in all cases obtained from the analysis of variance (ANOVA).

The accuracy of an analytical procedure is defined as the closeness of agreement between a result and its true value [24]. In the present study, the accuracy was examined by analyzing placebo samples spiked at three concentration levels (Low, Mid and High), corresponding to 8.0, 32.0 and 64.0 μg mL^−1^. Three individual replicates for each sample were carried out. The accuracy data are presented in Table 2. The excipients’ matrix did not interfere with the DSP analysis since the percent recoveries (%R) ranged between 97.4% and 101.4%. These results are in accordance with the requirements of international pharmaceutical guidelines [28].

### 3.4. Dissolution Test

**Figure 5:**
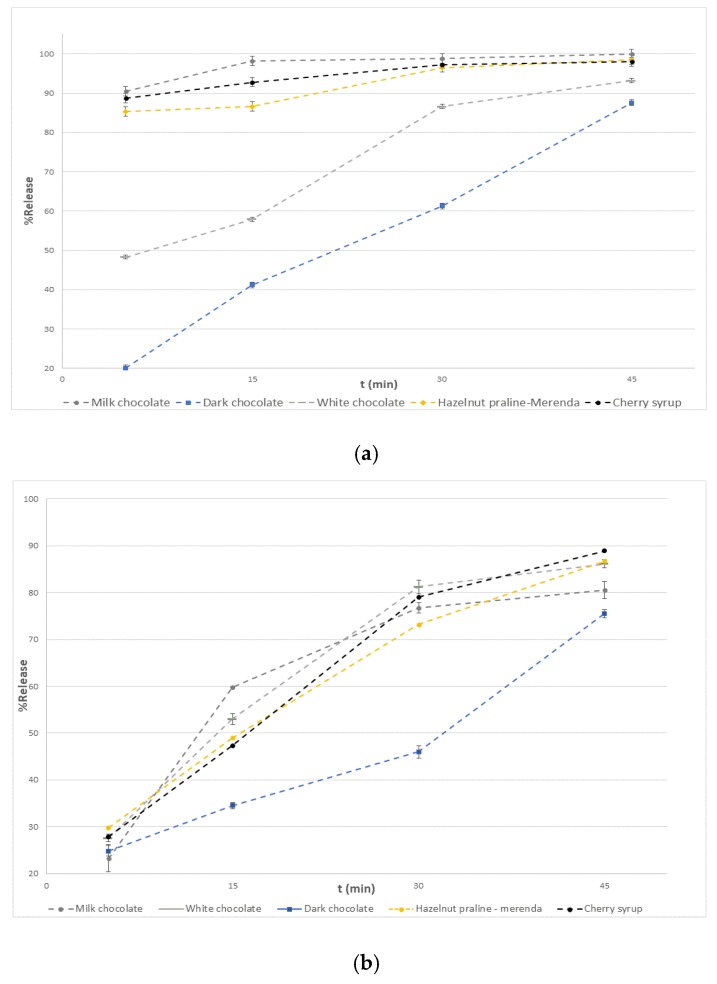
(**a**) Dissolution profiles of all formulations without centrifugation before extraction procedure, (**b**) dissolution profiles of all formulations after centrifugation before extraction procedure.

All dissolution studies were performed using a USP dissolution apparatus (type 2) in a manual-sampling dissolution bath (Vankel, VK 7010, Varian, Palo Alto, CA, USA). A volume of 500 mL of 1% *v*/*v* HCl aqueous solution was used as a dissolution medium while the stirring was set at 100 rpm. The temperature of the water bath was kept constant at 37 ± 0.5 °C. The acceptance criterion was established at 70% of the labeled amount of API, released in 45 min. [29]. Each formulation was crushed evenly before dissolution to simulate mastication by a young patient. An aliquot of 2 mL of sample was withdrawn (without medium replacement) at time intervals of 5, 15, 30 and 45 min. Then, a volume of 1000 μL of acetonitrile (or 2000 μL in the case of hazelnut praline formulations) was added and the mixture was centrifuged at 4500 rpm for 5 min. It should be noted that CH_3_OH (1000 μL) was used instead of acetonitrile for the pre-treatment of syrup-containing formulations. The samples were left in the freezer for 15 min and then analyzed by the proposed HPLC method. Three preparations for each formulation (2 mg API) were made, and the results are depicted in Figure 5a,b.

According to the results (Figure 5b), DSP is sufficiently released from all substrates at the end of the experiment at a time range of up to 45 min. Moreover, the dark chocolate preparations present a significant delay up to 30 min compared to the other matrixes. This imbalance is restored in the remaining 15 min. One possible interpretation of this phenomenon is that its composition at cocoa solid and cocoa butter (35% cocoa liquor) is superior than the other types of chocolate, making it more compact, while the protein content is lower. Indeed, after checking the hardness of different types of chocolate, dark chocolate proved to be harder (see Table 3).

**Figure 6:**
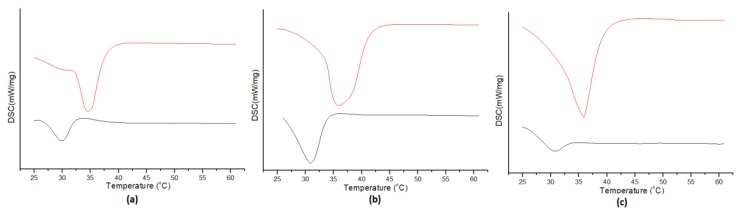
DSC diagrams: (**a**) milk chocolate, (**b**) dark chocolate, (**c**) white chocolate (black lines) and their drug-loaded formulations (red lines).

In addition, the DSC charts (Figure 6) show that the melting point of the dark chocolate is about 1 degree higher than the rest and almost the same as the operating temperature of the dissolution device (37 °C). Thus, its fat components encapsulate the lipophilic active ingredient of DSP for a longer time and keep it in suspension in the dissolution medium. Finally, comparing the two shapes, we concluded that at the end of the experiment (45 min), all the amount of the active substance is in the suspension while about 10% of it remains conjugated with the different types of chocolate. At this point, it should be clarified that DSP is trapped in the suspension and not in the precipitate, since in a parallel experiment which was carried out with the same procedure (but without centrifugation of the samples), the total amount of DSP (conjugated and free) presented in the suspension was determined at higher levels in the respective time points.

### 3.5. In Vitro Simulation of Gastrointestinal Digestion

In order to monitor the fate of added DSP in chocolate matrixes, an in vitro digestion model was adopted, according to the standardized static in vitro digestion method for food as is proposed in Reference [30]. In vitro models that simulate digestion processes are a useful tool to study the behavior of food or pharmaceuticals across the gastrointestinal tract (GIT). All in vitro models are advantageous in terms of time consumption, overall cost, labor intensity and ethical restrictions since simultaneous measurements of larger numbers of samples are allowed. Other advantages of the in vitro digestion models are the reproducibility, the control over the process conditions and the facile sampling at the sites of interest according to the needs of the study. Therefore, in vitro digestion models are considered the preferred tool for mechanistic studies and hypothesis testing [30].

#### 3.5.1. Experimental Procedure

Two in vitro digestion phases (an oral phase and a gastric phase) were performed as follows:

Oral Phase: chocolate samples were mechanically broken down with a blade and a specified amount of sample was weighed and placed in a 50 mL centrifuge tube. Simulated Salivary Fluid (SSF, pH 7) at 37 °C, was added to the tubes at a 1:1 (*v*/*w*) ratio. The system was homogenized and incubated for 2 min at 37 °C. Human α-amylase was added in a final concentration of 75 U/mL while samples without α-amylase were used as a control sample. The recommended time of contact with the enzyme is 2 min at 37 °C, which requires the pre-warming of all reagents to 37 °C.

Gastric Phase: following the oral phase, simulated gastric fluid (SGF, pH 3) was added to the tubes containing the oral bolus (1:1 *v*/*w*). Porcine pepsin was added into the SGF at a final concentration in the gastric mixture of 2000 U/mL. The pH of the mixtures was adjusted to pH 3 ± 0.1 using 1 N HCl and samples were placed at 37 °C, under constant mixing (using a rotary mixer). The reaction was terminated after 2 h by raising the pH value to 8 and immediately placing the samples at −20 °C.

Each experimental condition was performed in triplicate.

#### 3.5.2. DSP’s Extraction Procedure

The samples were withdrawn from the freezer and left at room temperature. Each sample was centrifuged for 45 min at 4500 rpm in order to separate the two phases (particle size studies were carried out in aqueous phase). Thereafter, the aqueous phase and sediment were transferred into separate centrifuge tubes and water (1.7 mL in the case of aqueous phase and 3.5 mL in the case of sediment) was added. Each mixture was placed into an ultrasound bath for 15 min and then it was centrifuged for 5 min at 4500 rpm. Subsequently, 1 mL of each supernatant fluid was transferred into new centrifuge tubes and 1.5 mL of acetonitrile was added in order to precipitate the proteins. The mixtures were sonicated for 15 min, centrifuged for 5 min at 4500 rpm and they were left in the refrigerator for half an hour. Finally, 1 mL of each supernatant was further mixed with 1 mL of acetonitrile and the same procedure was performed. The new supernatants were collected in vials and injected into HPLC.

#### 3.5.3. Particle Size Studies

**Figure 7:**
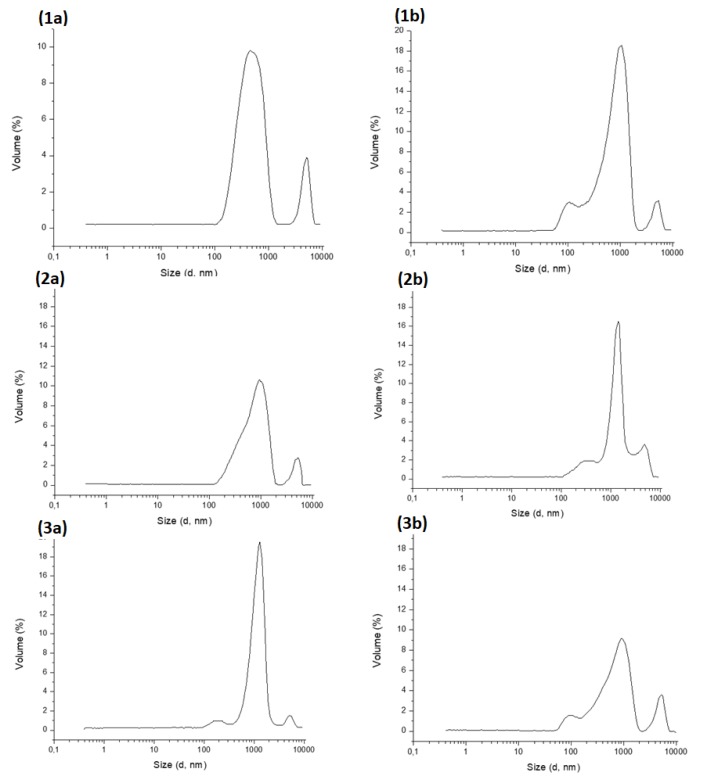
The dynamic light scattering (DLS) footprints from samples from empty and drug-loaded formulations in distilled water (**a**) and simulated gastric fluid SGF pH 3 (**b**): (1) milk chocolate, (2) white chocolate, (3) dark chocolate.

The dynamic light scattering (DLS) footprints of samples from empty and drug-loaded formulations in distilled water and SGF pH 3 are shown in Figure 7. The experimental data yielded bimodal distributions of particle sizes in digested water, where the hydrodynamic diameter of the majority of the smaller particles (first peak) lies in the region of 700 nm and the larger particles in the region of 7 μm.

In distilled water, the particle size distribution consists of two distinct regions of small and large particles respectively, as was mentioned before. On the other hand, in SGF, the particle size distribution of the small particles is shifted towards a larger hydrodynamic diameter in the region of 1000 nm. This shift is observed for all three types of chocolate: dark, milk and white chocolate, the last two having a more intense shift compared to the dark chocolate. This increase of the mean hydrodynamic radius of the smaller particles is attributed to the protein’s agglomeration in the acidic environment of the gastric phase [31]. Both milk and white chocolate have higher amounts of milk solids, and subsequently, higher amounts of milk protein in contrast to dark chocolate, in this way explaining the more intense shifting of the particle size distribution.

The reported polydispersity index (PI) values, ranging from 0 (ideally monodispersed) to 1 (system with very broad size distribution), were between 0.314 up to 0.635, respectively. In accordance with expectation, chocolate carriers possess negative charge in the range of ‒13 mV to ‒15 mV due to the presence of cocoa butter [32]. Consistent with the presence of adsorbed DSP on the surface of chocolate particles, the ζ-potential of all formulations tested shifted towards slightly higher values (not significantly different, *t*-test, p > 0.05) compared to their plain congeners, which might be attributed to the presence of the API on the surface of the carriers, as illustrated in Table 4. A significant increase towards higher ζ-potential values when compared with those obtained in digested water was recorded in SGF. The effect of pH on ζ-potential of the particles can be quantitatively explained by just assuming that an increase in H^+^ adsorption on the particles’ surface is taking place. Such adsorption would result in decreasingly negative surface charge on the particle surface, as the pH value of the surrounding is strongly acidic.

#### 3.5.4. In Vitro Digestion

**Figure 8:**
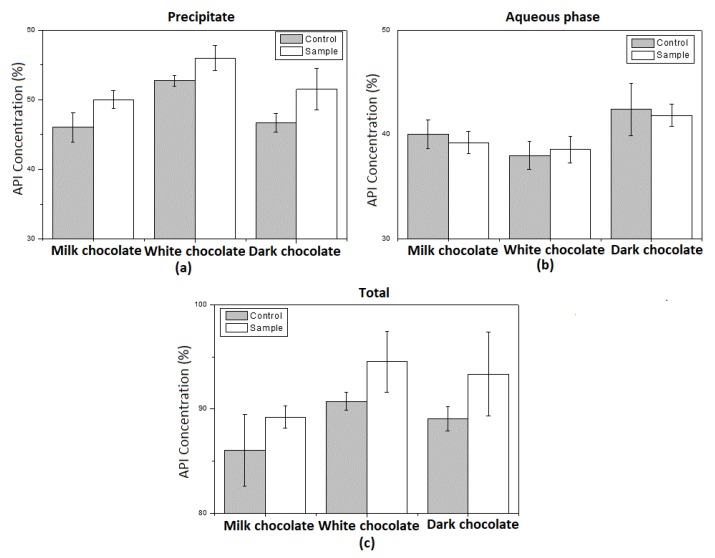
In vitro digestion: Amount of DSP recovered from (**a**) the aqueous phase, (**b**) the water insoluble phase and (**c**) the total of the two.

**Figure 9:**
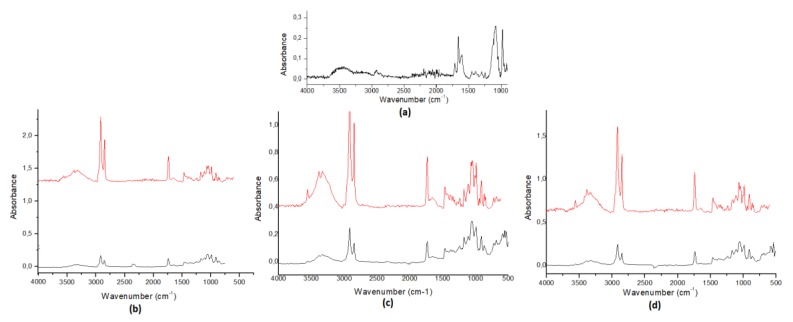
Fourier-transform infrared spectroscopy (FT-IR) diagrams: (**a**) DSP, (**b**) milk chocolate, (**c**) dark chocolate, (**d**) white chocolate (black lines) and their drug-loaded formulations (red lines). Band at 800–1000 cm^−1^ is characteristic of P–O bending mode (DSP). The wide band at 960–1100 cm^−1^ were assigned to C–O and C–C stretching modes, while bands at 1200–1500 cm^−1^ are associated with O–C–H, C–C–H, C–O–H and phosphate bending modes. The wide band at 3100–3500 cm^−1^ was assigned to O–H stretching. Most of the additional bands in the spectrum of chocolate were associated with lipids, which are consistent with the emulsion, while other bands were assigned to carbohydrates.

The fate of DSP mixture with various types of chocolate across the gastro-intestinal tract was simulated by an in vitro digestion model. Figure 8 depicts the amount of DSP recovered from the aqueous phase, the water insoluble phase and the total of the two. The results show that for all three types of chocolate (milk, white and dark), the amount of DSP in the aqueous phase was not affected by the enzymes present in the oral and gastric digestion phase (control versus sample). Additionally, there is a small increase in the total amount of DSP in the aqueous phase for dark chocolate, compared to the other types of chocolate. The concentration of DSP in the water insoluble phase is clearly affected by the action of the enzymes present in the digestion process. The amount of the API in the digested water insoluble phase is higher than the amount in the control undigested sample. This is an indication of the association of the DSP with the polysaccharide moiety of the insoluble phase [33,34]. Finally, the total recovery of DSP from the digestion media is less than 95% (Figure 9), indicating the association of the API with the insoluble fraction of chocolate, as explained previously.

### 3.6. Stability Study

#### 3.6.1. Stability of DSP During the Manufacturing Process

**Figure 10:**
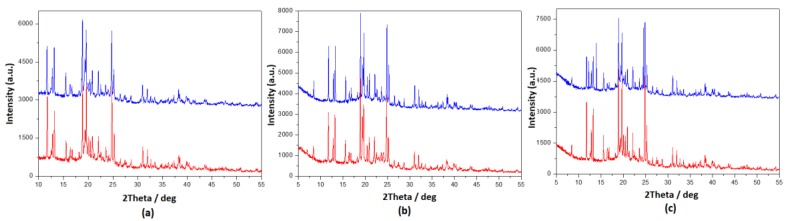
X-ray diffraction (XRD) diagrams of (**a**) milk chocolate, (**b**) dark chocolate and (**c**) white chocolate without DSP (blue lines) and of drug-loaded formulations (red lines).

Dark, white and milk or stevia chocolate was used for the manufacturing process, while hazelnut chocolate praline and syrup were convenient for creating in-house preparations. Because of the fact that both matrix and API were pretreated at 60 °C during the manufacturing process, their stability was tested using differential scanning calorimetry (DSC) (Figure 7), Fourier-transform infrared spectroscopy (FT-IR) (Figure 9) and X-ray diffraction (XRD) techniques (Figure 10). Spectra from placebo and drug-loaded formulations were recorded, and their results were evaluated. According to the resulting data, DSP is affected neither by the matrix nor from the relatively high temperature (60 °C) since its peak’s area and profile remain stable.

#### 3.6.2. Long-Term Stability

**Figure 11:**
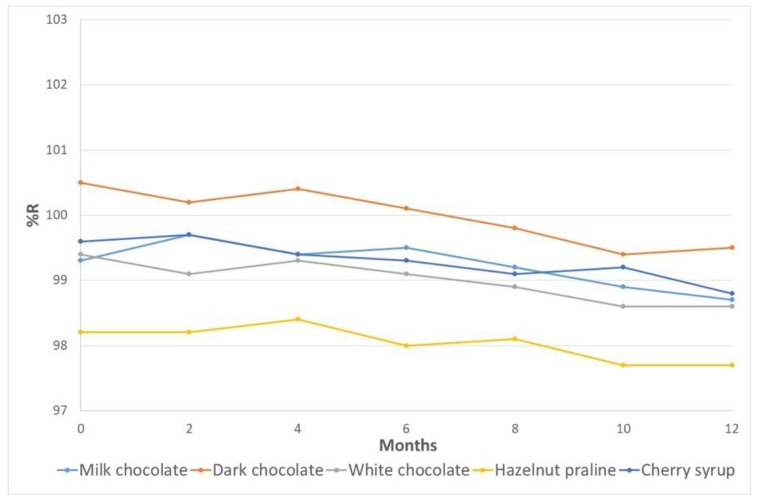
Stability test for the new formulations over a period of 12 months.

The stability of DSP in the prepared edible formulations was examined for up to 1 year, and were kept at 4 °C. Six different preparations for each formulation were made. The obtained recoveries ranged from 97.7% to 100.5%. It was found that the DSP concentration in the stored samples was less than 5% compared to the freshly prepared samples, confirming the stability of the DSP drug in the studied matrixes. Figure 11 represents the stability of the DSP over the examined period of time.

## 4. Conclusions

The new chewable formulations were devised on purpose to improve the acceptability and palatability of its API. Upon successful completion of this study, parents will be able to administer the steroid to their children by giving them factory-prepared chocolate (dark, milk, milk with sweeteners or white chocolate, and with strawberry/cherry syrup or with hazelnut praline). In order to verify the acceptability of chocolate as a carrier for the drug, once the respective pharmaceutical formulations were formulated, both their active content and their stability (at 4 °C) over a period of 12 months were checked.

Therefore, a validated routine analysis method was developed to carry out the study, taking into consideration the requirements outlined in ICH guidelines.

The current study detailing easy-to-consume formulations for special populations merits further investigation in a hospital setting for their in vivo evaluation. 

## Figures and Tables

**Table 1:** Quantities of excipients’ and dexamethasone sodium phosphate (DSP) per formulation or per daily dose.

DSP/Formulation (mg)	DSP/Day (mg) *	PG/Formulation (mg)	PG/Day (mg) *	Chocolate/Formulation (g)
1.0	2.0	155.3	310.5	4.0
2.0	4.0	310.5	621.0	4.0

* 1 formulation/12 h. DSP: Dexamethasone sodium phosphate, PG: Propylene glycol

**Table 2:** Accuracy data for active pharmaceutical ingredient (API) (spiked placebo samples).

Sample		Milk Chocolate		Dark Chocolate		Milk Chocolate (Stevia)		White Chocolate		Hazelnut Praline (Merenda)		Strawberry Syrup		Cherry Syrup	
Spiked Placebo	Spiking Level (μg/mL)	Amount of API Found (μg/mL)	Recovery (%)	Amount of API Found (μg/mL)	Recovery (%)	Amount of API Found (μg/mL)	Recovery (%)	Amount of API Found (μg/mL)	Recovery (%)	Amount of API Found (μg/mL)	Recovery (%)	Amount of API Found (μg/mL)	Recovery (%)	Amount of API Found (μg/mL)	Recovery (%)
Sample 1	64.0	63.9	99.8	63.6	99.3	63.4	99.1	63.0	98.5	62.6	97.8	62.7	97.9	63.5	99.2
Sample 2	64.0	64.6	100.9	64.7	101.1	64.7	101.1	63.3	98.9	63.6	99.3	63.1	98.6	63.1	98.6
Sample 3	64.0	64.5	100.8	63.3	98.9	64.5	100.8	64.2	100.3	62.7	98.0	62.8	98.1	63.3	98.9
Sample 1	32.0	31.7	99.0	32.3	100.8	32.4	101.1	31.7	99.1	31.2	97.4	31.3	97.8	31.5	98.5
Sample 2	32.0	32.0	100.1	32.2	100.5	31.6	98.6	32.3	101.0	32.3	100.9	31.3	97.9	31.7	99.2
Sample 3	32.0	32.3	101.1	31.7	99.2	31.7	99.2	32.4	101.4	31.8	99.4	32.0	99.9	31.6	98.9
Sample 1	8.0	7.9	98.6	7.8	98.1	7.9	99.3	7.8	98.0	8.1	100.8	8.0	99.4	7.9	98.6
Sample 2	8.0	8.0	100.2	7.9	99.3	8.0	99.8	7.9	98.4	7.9	98.9	7.9	98.6	8.0	99.6
Sample 3	8.0	7.9	99.0	8.0	99.4	8.1	100.9	7.9	99.1	8.0	99.5	7.9	98.9	7.9	98.9
		Mean	99.9	Mean	99.6	Mean	100.0	Mean	99.4	Mean	99.1	Mean	98.6	Mean	98.9
		%RSD	0.9	%RSD	0.9	%RSD	0.9	%RSD	1.1	%RSD	1.2	%RSD	0.7	%RSD	0.3

%R: Percent recovery, %RSD: Percent relative standard deviation.

**Table 3:** Quality control tests—physicochemical properties.

Type of Chocolate	Hardness * (kp)	Friability **	Disintegration Time *** (min)
Milk chocolate	8.8	Loss of mass = 0.2%	-
Milk chocolate + DSP	5.9	Loss of mass = 0.3%	20
Milk chocolate (stevia)	8.5	Loss of mass = 0.2%	-
Milk chocolate (stevia) + DSP	5.7	Loss of mass = 0.3%	22
Dark chocolate	9.2	Loss of mass = 0.1%	-
Dark chocolate + DSP	6.3	Loss of mass = 0.2%	36
White chocolate	7.6	Loss of mass = 0.2%	-
White chocolate + DSP	4.0	Loss of mass = 0.5%	24

* 3 replications in each case. ** 10 samples/formulations were tested in each case (European Pharmacopeia 2.9.7). *** 6 samples/formulations were tested in each case (Ph. Eur. 2.9.1)/set temperature: 37 ± 0.5 °C.

**Table 4:** ζ-potential values of chocolate dispersions.

Formulation	*ζ*-Potential ± Standard Deviation (mV) in Distilled Water	*ζ*-Potential ± Standard Deviation (mV) in SGF
Milk Chocolate	−13.5 ± 0.0	−8.2 ± 17.1
Milk Chocolate + DSP	−11.4 ± 9.37	−9.0 ± 27.7
White Chocolate	−14.0 ± 21.6	−9.9 ± 21.8
White Chocolate + DSP	−13.6 ± 11.2	−9.8 ± 8.6
Dark Chocolate	−15.3 ± 13.7	−12.7 ± 14.3
Dark Chocolate + DSP	−14.9 ± 9.3	−11.7 ± 14.1

## Supplementary Materials

The following are available online at https://www.mdpi.com/1999-4923/12/4/354/s1, Figure S1: Schematic diagram of sample pre-treatment and extraction unsuccessful efforts of chocolate and other (hazelnut praline and tea-spoon syrup) formulations.
