# Supplementary material for: Solid Dosage Forms of Dexamethasone Sodium Phosphate Intended for Pediatric Use: Formulation and Stability Studies
Source: Pharmaceutics. 2020 Apr 14;12(4):354. doi: 10.3390/pharmaceutics12040354 (PMC7238162; doi:10.3390/pharmaceutics12040354)
Supplement: Supplementary file 1 [file pharmaceutics-12-00354-s001.pdf]

pharmaceutics

Supplementary Materials: Solid Dosage Forms of Dexamethasone Sodium Phosphate Intended for Pediatric Use: Formulation and Stability Studies

Maria S. Synaridou, Eleftherios G. Andriotis, Constantinos K. Zacharis, Dimitrios G. Fatouros and Catherine K. Markopoulou

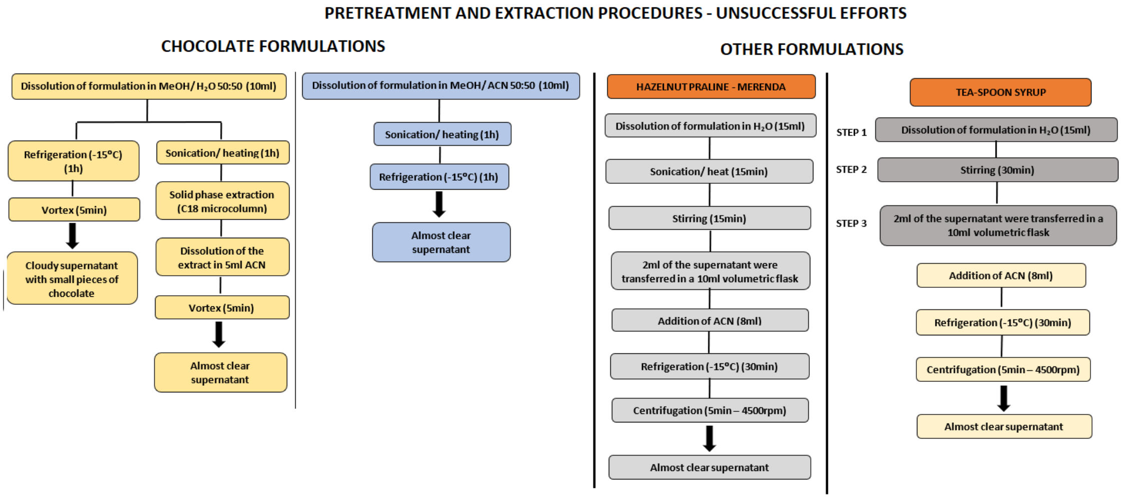

Figure S1. Schematic diagram of sample pre-treatment and extraction unsuccessful efforts of chocolate and other (hazelnut praline and tea-spoon syrup) formulations.
